# Genetic Parameters of Egg Quality Traits and Albumen Density in White Leghorn Chickens

**DOI:** 10.3390/ani16020284

**Published:** 2026-01-16

**Authors:** Anqi Chen, Haiyan Wang, Dengjing Zuo, Haiying Li, Huie Wang, Zhonghua Ning, Liping Ban, Changqing Qu, Xiaoyu Zhao, Lujiang Qu

**Affiliations:** 1 National Engineering Laboratory for Animal Breeding, College of Animal Science and Technology, China Agricultural University, Beijing 100193, China; 18800017599@163.com (A.C.); 13324944209@163.com (D.Z.); ningzhh@cau.edu.cn (Z.N.); 2 Hohhot Customs District, Hohhot 010010, China; why20042006@126.com; 3 College of Animal Science, Xinjiang Agricultural University, Urumqi 830000, China; lhy-3@163.com; 4 Key Laboratory of Tarim Animal Husbandry Science & Technology, College of Animal Science and Technology, Tarim University, Alar 843300, China; whedky@126.com; 5 College of Grassland Science and Technology, China Agricultural University, Beijing 100193, China; lipingban@cau.edu.cn; 6 Engineering Technology Research Center of Anti-Aging Chinese Herbal Medicine of Anhui Province, Biology and Food Engineering School, Fuyang Normal University, Fuyang 236037, China; qucq518@fynu.edu.cn; 7 Xingrui Agricultural Stock Breeding, Baoding 072550, China

**Keywords:** white leghorn, heritability, albumen density, protein content

## Abstract

The conventional method for detecting protein content in egg albumen is the Kjeldahl method, but this method cannot be applied in practical production due to cost limitations. Therefore, we developed albumen density (AD), which had certain potential application value in low-cost and efficient evaluation of albumen protein content. We calculated the heritability of AD in White Leghorn (WL) chickens and its correlation with average albumen protein quantity (AAP), total albumen protein quantity (TAP), albumen weight (AW), albumen volume (AV), egg weight (EW), albumen height (AH), haugh unit (HU), and yolk color (YC). It is worth noting that albumen protein content was measured in a small subset of samples. The average value of AD in eggs was 0.97 and its heritability was less than 0.1. The average value of AAP in eggs was 10.1%, and the average value of TAP in eggs was 2.95 g. There were significant positive correlations between AAP, TAP, AW, AV, AD, and EW, and there were strong positive genetic and phenotypic correlations between EW, AW, AV, and AD. The results of this study indicated that AD might have potential value as a supplementary tool for albumen protein trait selection in breeding.

## Simple Summary

In practical production, the traditional Kjeldahl method was not suitable for large-scale use to determine the protein content of eggs, which was a key issue. Therefore, our research aimed to seek an indicator that could be used to estimate the albumen protein content in different types of eggs. Our study measured the albumen weight (AW) and albumen volume (AV), and then calculated albumen density (AD). In addition, we accurately measured the average albumen protein quantity (AAP) and total albumen protein quantity (TAP) in one batch of eggs using the Kjeldahl method. The results showed highly significant positive correlations between AD, AAP, and TAP, indicating that we could use this indicator to estimate protein content.

## 1. Introduction

Eggs are the most common and important dietary component in people’s daily lives; they are loved by people for their low price and high nutritional value [1,2]. Egg yolk and albumen are the most usable parts of eggs, containing a rich variety of amino acids, which are important components in providing protein in eggs [2,3,4,5,6]. The high digestibility and amino acid balance of egg yolk and albumen make them a source of high-quality protein for the human body [7]. And the presence of vitamins, selenium, and other antioxidants in eggs makes them an important antioxidant food [8], and the addition of vitamin D3 to feed can affect its content in eggs [9]. However, various factors such as egg laying age [10,11], chicken breed [5,6,12,13,14,15], feed additives [16,17,18,19], feed type [6], and housing system [20,21] can affect the nutritional contents of eggs.

The increasing demand for consumer choices guides eggs and their products towards diversification [22], such as specialty foods like green shell eggs [23,24]. The diversification of processing methods also ensures the quality of eggs used in food processing, such as commercial processing for modification and cleaning, which helps enhance eggshell function [25] and reduces microbial contamination [26]. The quality and freshness of eggs are generally evaluated using Haugh unit (HU) in production [22], which decreases during storage and are influenced by various factors such as storage time [27,28,29,30,31] and storage conditions [32,33,34,35,36,37,38]. And the decrease in egg quality also means a decrease in protein quantity [28]. In addition, albumen gel properties are also indicators for evaluating the quality of eggs based on appearance [39,40], including changes in gel ability, antibacterial activity, viscosity, elasticity, and protein contents of albumen during storage [41]. In general, long-term storage of eggs can cause the albumen to become thinner and eventually turn into liquid, resulting in a decrease in the quality, viscosity, and protein contents of the eggs [42]. Therefore, the protein contents in albumen are an important factor related to improving the quality of eggs. The Kjeldahl method was commonly used to measure the protein contents in eggs [4], but the time and monetary costs associated with this method also mean that its use in breeding was greatly limited. Given the limitations of traditional methods, we planned to develop an indicator with protein evaluation application value in breeding. We speculated that there might be a correlation between albumen density and albumen protein content, but there is little research in this area.

## 2. Materials and Methods

### 2.1. Animals and Egg Collection

More than 2000 healthy White Leghorn (WL) chickens at 45 weeks of age and raised in the same housing conditions were selected for egg collection. All chickens were raised using a 3-tiered step single-cage feeding system and were fed according to the same feeding standards. Starting from day 413, we collected all eggs from each chicken for 3 consecutive days, and the weight and quality of normal eggs collected for 2 consecutive days were all tested within 72 h after they were sent to the laboratory. The temperature in the storage environment was 18–20 °C.

### 2.2. Measurements of Egg Quality and Albumen Density

In order to ensure measurement repeatability, reduce measurement errors, and improve measurement efficiency, all egg measurements were completed by the same person, and data recording was the responsibility of another person. However, due to unavoidable factors such as measurement time and machine errors, potential measurement errors might exist. There was no preference for the order in which each egg was measured, which meant that egg measurements were randomly sampled, and under this rule, we measured all eggs. Egg weight (EW) was measured with an electronic scale and the result was accurate to 0.01 g. Eggshell strength (ESS) was measured with an eggshell force gauge (MODEL-III, Robotmation Co., Ltd., Tokyo, Japan). Subsequently, the eggs were broken and the internal contents were transferred to a multifunctional egg tester (EMT-7300, Robotmation Co., Ltd., Tokyo, Japan) to measure albumen height (AH), haugh unit (HU), and yolk color (YC). Then we used a yolk separator to separate the contents, measured the yolk weight (YW) and albumen weight (AW) using an electronic scale, and measured the albumen volume (AV) using a 100 mL graduated cylinder. Albumen density (AD) was the ratio of AW to AV. Albumen percentage (AP) was the ratio of AW to EW. Yolk percentage (YP) was the ratio of YW to EW. Yolk/Albumen (YA) was the ratio of YW to AW. After obtaining the egg quality measurement data, we observed that some of the egg measurement values were abnormal, but we did not filter them because we believed they were within the normal error range in the experiment, as mentioned earlier.

### 2.3. Measurements of Protein Contents in Egg Albumen

In order to discover the relationship between AD and average albumen protein quantity (AAP), we conducted a preliminary experiment by using the same method as described above to randomly detect 31 WL eggs laid on the same day from the same population and collected the albumen of each egg. Subsequently, the albumen was homogenized for AAP determination. AAP was calculated from nitrogen content determination by the Kjeldahl method, and the conversion factor from nitrogen to protein was 6.32. And total albumen protein quantity (TAP) was calculated based on AAP and sample quantity. The above analysis was only a preliminary verification.

### 2.4. Genetic Parameter Estimation

Based on complete phenotype and pedigree data, we used univariate and multivariate model in DMU V6 R5.2 software [43] to estimate the genetic parameters of AD and other traits, as shown below.

y = Xμ + Za + e(1)

In this model, y is the phenotypic observations of traits, X is relation matrix of fixed effects, μ is vector composed of fixed effects, Z is correlation matrix of random effects, a is vector composed of random effects, and e is random residuals.

### 2.5. Statistical Analysis

Descriptive statistics and Pearson correlation analysis in pre-experiment were conducted using SPSS software (version 26.0; IBM Corp., Armonk, NY, USA).

## 3. Results

### 3.1. Egg Quality and Albumen Density of WL Chickens

The descriptive statistics of AD and egg quality traits are shown in Table 1 and Table 2. The average value of AD measured was overall around 0.97, and the average value of YC measured was overall around 0.72. In addition, we could see that the average and minimum values of HU and AH in Table 2 were lower than those in Table 1.

### 3.2. Protein Contents of Egg Albumen

The protein contents of 31 samples were determined using the Kjeldahl method, and the results are shown in Table 1. From the results, it could be seen that the AAP of albumen ranged from 9.21 to 11.1%, while the TAP of albumen ranged from 1.97 to 3.80 g, and the coefficient of variation of TAP was higher than that of AAP, indicating that the TAP was also related to EW. Then we conducted the correlation analysis on the test results of 31 eggs, and the results are shown in Table 3. The results showed significant positive correlations between AAP, TAP, AW, AV, AD, and EW, which meant that larger eggs might have more egg albumen and higher protein contents. AD was associated with AAP and TAP. But the applicability of this result was limited by the sample size.

### 3.3. Genetic Parameter Estimates

The genetic parameters of AD and egg quality traits estimated by univariate and multivariate model are shown in Table 2 and Table 4, respectively, and the results in both tables were consistent. In general, we found that the heritability of AW, AV, YW, EW, ESS, YC, YP, AP, and YA were all greater than 0.2, but the heritability of AD, AD, and HU were all less than 0.1. There were strong positive genetic and phenotypic correlations between EW, AW, AV, and AD, which also supported the results in Table 3. Therefore, we concluded that AD was less affected by genetic factors. However, as a derived trait, the correlation between AD and other traits might have mathematical dependence.

## 4. Discussion

### 4.1. Egg Quality Traits and Albumen Protein Contents

Eggs provide various essential nutrients for humans [2], among which protein and other components play certain functional characteristics and biological activities [1], making them an important source of high-quality protein and leading to their wide use in the food industry [7,44]. But under different conditions, egg protein might undergo potential changes, which could affect its nutritional value and biological activity [44]. However, egg albumen and albumen proteins have received less attention in other research. Our study focused on the correlations between egg quality traits, AD, and egg albumen protein content, providing a theoretical basis for the effective utilization of egg albumen. During the egg quality testing period, the average values of AV, AW, and EW in the pre-experiment period were lower than those in the formal testing period, which might be due to the use of eggs from younger hens in the pre-experiment period. In the formal experiment, we determined that the number of eggs was larger and the usage time was longer. The limitation of measurement time and inevitable machine errors were the main reasons for the abnormal HU values in Table 2 and the HU values being lower than those in Table 1. We used the Kjeldahl method to measure the average value of AAP was 10.1, and the average value of TAP was 2.95. Lordelo et al. (2020) found that the protein content of albumen in various types of chicken eggs was all around 10.5%, and there was no significant difference between them [45]. Shafer et al. (1996, 1998) also found that the protein content of chicken egg albumen was around 10.0%, but different levels of methionine intake had a significant impact on the protein content of egg albumen [46,47]. The range of crude protein content in the egg albumen of six types of poultry (chicken, duck, goose, turkey, pigeon, and quail) was 8–11%, with goose having the lowest protein content, followed by pigeons, and the highest being chickens and turkeys [48]. But on dry matter basis, the protein content of albumen in various types of eggs ranged from 78% to 86% [49]. However, Cendron et al. found that the protein content of eight types of egg albumen was 8–10% on a wet basis and 83–89% on a dry matter basis [50]. The difference between Cendron et al.’s results and Somes et al.’s results might be due to differences in chicken breeds and updated measurement methods. The above studies all supported the results of our study. However, due to the limitations of the sample size and traditional measurement methods in this study, it was necessary to continue large-scale sample collection and validation. In addition, we also found that AAP and TAP were all significantly positively correlated with AV, AW, and EW indicating that egg albumen content and protein content increased with the size of eggs. In general, the protein content in egg albumen was affected by egg types (traditional or organic) [51], breeds [15,45,48,49,50,52], and methionine intake [46,47]. And the results of egg albumen proteomics of five local chicken breeds showed that 189 egg albumen proteins existed and most of them had antibacterial function, and the protein abundance in egg albumen across different breeds was different [53], which deepened our understanding of egg albumen.

### 4.2. Egg Quality Traits and Albumen Density

In our experiments conducted on two groups of WL eggs from different environments and periods, we measured the average values of AD and YC to be around 0.97 and 7.2, indicating that our study strictly followed the measurement standards. This result suggested that the heritability of these traits appeared to be similar in different populations. However, it could not be ignored that factors such as feeding environment and the age of hens were also important factors that affected the phenotypes of these traits. However, there were certain differences in the two measurement results of AV, AW, EW, HU, AH, and AP, indicating that the age and feeding environment of hens, as well as the egg storage time, had a certain impact on egg quality traits. Kowalska et al. (2021) also found that the average value of thick albumen density (TAD) at different egg laying stages was overall at around 1.0, and there were significant differences in EW, AW, YW, AP, YP, HU, AH, TAD, and YC at different ages of hens, indicating that the egg quality and TAD changed with the aging of hens [54]. But in our study, we used the whole egg albumen to calculate AD. Therefore, the slight differences in AD between the above results and our study might be due to differences in measurement methods, egg albumen selection, and egg types, which also indicated that thick albumen might have had more components than other parts of egg albumen. And the effects of diets with different protein sources on HU, AH, and YC were also found to be significant [54]. In terms of egg quality traits, it might be affected by light stimulation [55,56], hen age [57,58], breed [22,45,59,60,61], heat stress [62], and feed additives [46,63,64,65,66,67,68,69].

Based on complete pedigree and phenotype data, we estimated that the heritability of AD was less than 0.1, and AD was significantly positive correlated with AW, AV, and EW. As mentioned in our discussion, the protein content in egg albumen increases with the increase in EW, and the molecular weight of protein was higher than that of water, which led to the increase in AD with the increase in EW. Our study was the first to reveal the genetic mechanism of AD, and there were few studies to estimate the heritability of AD and its correlation with production traits and egg quality traits. The heritability of yolk proportion and eggshell strength were all greater than 0.2 [70,71], and genetic selection was used to change yolk proportion [72,73] and improve eggshell quality [74]. Zhang et al. (2021) found seven candidate genes that affect the yolk proportion in Wenchang chickens, including MNR2, AOX1, ANTXRL, GRAMD1C, EEF2, COMP, and JUND [75], while Chen et al. discovered three candidate genes related to ESS in the pure line of Rhode Island Red, including FRY, PCNX2, and ENSGALG00000052468 [71]. However, the heritability of AD was relatively low, which indicated that the significance of the AD trait lay in phenotypic monitoring rather than genetic selection centered on it.

## 5. Conclusions

In general, egg quality and protein content of albumen were affected by many factors, and egg freshness declined with storage time. This study preliminarily explored the possibility of using AD values as an indirect indicator for comparing the protein content of albumen. We found significant positive correlations between AAP, TAP, AW, AV, AD, and EW, indicating that AD could be used to indirectly reflect the protein content of albumen.

## Figures and Tables

**Table 1:** Egg quality traits and protein contents of albumen in pre-experiment.

Trait	Egg Number	Minimum	Maximum	Mean ± SD	CV/%
Albumen weight (g)	31	21.4	35.2	29.2 ± 3.4	11.6
Albumen volume (mL)	31	22.0	36.0	29.7 ± 3.2	10.8
Albumen density (g/mL)	31	0.93	1.02	0.98 ± 0.02	2.0
Egg weight (g)	31	48.5	63.3	55.6 ± 4.1	7.4
Albumen height (mm)	31	4.40	7.5	5.98 ± 0.7	12.2
Haugh unit	31	65.4	88.7	78.3 ± 5.4	6.9
Yolk color	31	5	10	7.19 ± 1.6	22.2
Albumen percentage (%)	31	43.9	58.2	52.4 ± 3.0	5.7
Average albumen protein quantity (g/100 g)	31	9.21	11.1	10.1 ± 0.6	5.9
Total albumen protein quantity (g)	31	1.97	3.80	2.95 ± 0.4	13.6

SD is standard deviation.

**Table 2:** Egg quality traits of White Leghorn.

Trait	Sample Size	Minimum	Maximum	Mean ± SD	CV/%	h^2^ ± SE
Albumen weight (g)	1997	22.2	42.8	32.3 ± 2.84	8.79	0.37 ± 0.05
Albumen volume (mL)	1997	19.3	43.6	33.1 ± 2.82	8.52	0.35 ± 0.05
Albumen density (g/mL)	1997	0.7	1.48	0.97 ± 0.03	3.09	0.01 ± 0.03
Yolk weight (g)	1997	9.4	28.9	18.1 ± 1.86	10.28	0.28 ± 0.05
Egg weight (g)	1997	46.0	73.9	60.7 ± 3.88	6.39	0.37 ± 0.05
Eggshell strength	1997	1.22	5.41	2.81 ± 0.63	22.2	0.16 ± 0.04
Albumen height (mm)	1997	1.4	13.6	4.94 ± 1.41	28.5	0.03 ± 0.03
Haugh unit	1997	19.2	111.8	66.0 ± 11.6	17.6	0.03 ± 0.03
Yolk color	1997	4.25	10.2	7.2 ± 0.62	8.61	0.20 ± 0.04
Yolk percentage (%)	1997	15.7	46.6	29.9 ± 2.54	8.49	0.28 ± 0.05
Albumen percentage (%)	1997	42.1	66.2	53.2 ± 2.56	4.81	0.31 ± 0.05
Yolk/Albumen (%)	1997	29.7	91.2	56.4 ± 6.62	11.74	0.36 ± 0.05

The h^2^ is heritability, which is estimated using univariate model, SD is standard deviation, SE is standard error.

**Table 3:** The correlation between protein contents and egg quality in pre-experiment phase.

Trait	AAP	TAP	AW	AV	AD	EW	AH	HU	YC
AAP	1								
TAP	0.760 **	1							
AW	0.514 **	0.947 **	1						
AV	0.494 **	0.933 **	0.991 **	1					
AD	0.357 *	0.501 **	0.497 **	0.378 *	1				
EW	0.499 **	0.888 **	0.925 **	0.912 **	0.497 **	1			
AH	0.141	0.169	0.155	0.139	0.196	0.130	1		
HU	0.063	−0.027	−0.066	−0.079	0.072	−0.074	0.958 **	1	
YC	−0.134	−0.037	0.013	−0.005	0.108	−0.009	0.307	0.281	1

Abbreviations: AAP = Average albumen protein quantity (g/100 g); TAP = Total albumen protein quantity (g); AW = Albumen weight (g); AV = Albumen volume (mL); AD = Albumen density; EW = Egg weight (g); AH = Albumen Height (mm); HU = Haugh unit; YC = Yolk color. * Represents significant difference at *p* < 0.05. ** Represents significant difference at *p* < 0.01.

**Table 4:** Estimation of genetic parameters for egg quality traits.

Trait	EW	AW	AV	AD
EW	0.37 (0.05)	0.88	0.86	0.99
AW	0.85	0.37 (0.05)	0.99	0.92
AV	0.81	0.95	0.33 (0.05)	0.90
AD	0.17	0.23	−0.09	0.02 (0.03)

The values on the diagonal in this table are the heritability of each trait estimated using a multivariate model and the values in upper and lower triangles are genetic and phenotypic correlations, respectively. SE values are present in parentheses. Abbreviations: AW = Albumen weight (g); AV = Albumen volume (mL); AD = Albumen density; EW = Egg weight (g).

## Data Availability

No sequencing data generated.
